# Understanding the Learning Curve of Carpal Tunnel Release With Ultrasound Guidance: A Review

**DOI:** 10.7759/cureus.41938

**Published:** 2023-07-15

**Authors:** Seper Ekhtiari, Mark Phillips, Dalraj Dhillon, Ali Shahabinezhad, Mohit Bhandari

**Affiliations:** 1 Orthopaedic Surgery, Addenbrooke's Hospital, Cambridge University Hospitals NHS Foundation Trust, Cambridge, GBR; 2 Health Research Methodology, McMaster University, Hamilton, CAN; 3 Faculty of Health Sciences, McMaster University, Hamilton, CAN; 4 Family Medicine, University of Ottawa, Ottawa, CAN; 5 Orthopaedic Surgery, McMaster University, Hamilton, CAN

**Keywords:** learning curve, minimally invasive, ultrasound guidance, carpal tunnel release, carpal tunnel syndrome

## Abstract

Carpal tunnel syndrome (CTS) is the most common compressive neuropathy and can be treated through carpal tunnel release (CTR) if nonoperative treatments fail. CTR can be performed through a variety of techniques, including traditional open, mini-open, endoscopic, and CTR with ultrasound guidance (CTR-US). The evidence on endoscopic CTR is mixed, due to a higher potential for nerve injury with endoscopic CTR compared to traditional open CTR. CTR-US offers the potential advantage of allowing the visualization of all key anatomical structures, combined with a very small incision and minimal soft tissue insult. As with any ultrasonographic technique or procedure, the learning curve needs to be considered for any provider considering adopting CTR-US. However, literature on ultrasound use around the wrist, including early evidence on the learning curve of CTR-US specifically, demonstrates this skill can be learned relatively quickly by providers with a wide range of prior experience in ultrasound and CTR. Overall, there is a need for high-quality studies comparing different CTR techniques, particularly CTR-US, as it offers the potential for considerable cost savings.

## Introduction and background

Carpal tunnel syndrome (CTS) is the most common compressive neuropathy [1]. In carefully selected patients who have failed nonsurgical management, surgical carpal tunnel release (CTR) is the standard of care, with nearly 600,000 procedures performed annually in the United States [2]. Although generally successful, up to 20% of patients have persistent symptoms following CTR [3]. Variable rates of revision surgical procedures have been reported, ranging from less than 1% to 12% [4]; most studies report revision rates between 0.5% and 1.5% [5]. While traditional open CTR is the most common surgical technique used to treat CTS, newer techniques, such as carpal tunnel release using real-time ultrasound guidance (CTR-US), may offer some advantages with accelerated recovery in the postoperative period [6]. As with any new technique, some potential adopters may have questions pertaining to the learning curve of the procedure [7].

## Review

The learning curve contextualized

**Figure 1:**
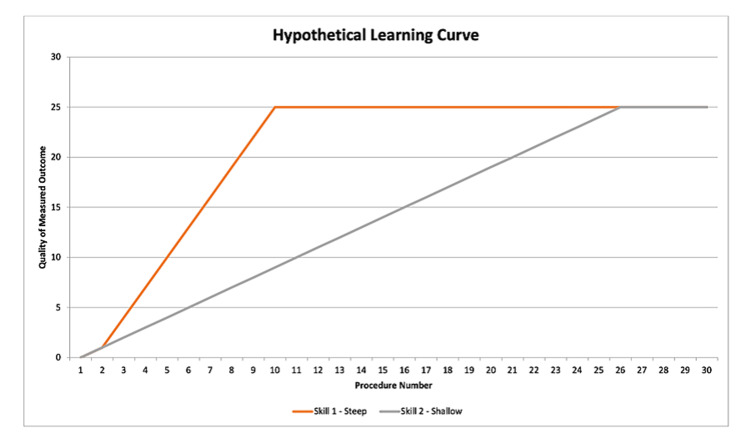
As displayed, a skill with a steeper learning curve is one that is mastered more quickly. This principle also holds true for skills where a lower value is desirable (e.g. blood loss) Figure property of the authors

Though the concept of the learning curve is generally familiar, it is worth defining it clearly in the surgical context. One of the earliest formal evaluations of the surgical learning curve was by Luft et al., who in 1979 reported on an association between the number of cases performed by a surgeon and lower mortality rates [8]. More recently, Hopper et al. characterized a typical surgical learning curve in four stages [9]: 1) a rapid ascent (i.e., improvement) in the measured outcome at the onset of training, 2) a zone of diminishing returns, where further experience only confers marginal improvements in outcome, 3) a plateau, where further experience has no additional benefit on the measured outcome, and 4) an age-related decline in the measured outcome. It is worth noting that the phrase “steep learning curve” may lead to confusion or misinterpretation. While commonly used to refer to a procedure or skill that takes a long time and/or many repetitions to master, this is not consistent with the visual representation of an actual learning curve. When plotting an outcome against the experience of the person performing it, a steeper learning curve actually applies to a skill that is more easily or quickly mastered (Figure 1) [10].

Multiple studies have documented the learning curves for a variety of ultrasound-guided procedures and demonstrated the ability of practitioners to achieve rapid proficiency. These include a wide range of procedures across various anatomical sites such as peripheral nerve localization, hip joint injection, and office-based detection of rotator cuff tears [11-14]. For example, sports medicine-trained orthopedic surgeons who were taught to perform and interpret shoulder ultrasounds became more accurate as they performed more scans, achieving up to 74% accuracy [13,15]. In addition, medical students plateaued in speed and accuracy following five repetitions of a peripheral nerve block procedure [16].

Injection of the hip joint, which is anatomically a deep structure surrounded by complex anatomy [17], including important neurovascular structures, has historically been considered to be a difficult skill and has been frequently deferred to radiologists to perform using image guidance. In recent years, however, orthopedic surgeons have increasingly started to perform ultrasound-guided injections of the hip, and a study by Plamen et al. has demonstrated that proficiency is achieved after only 30 cases [18]. Similarly, a one-day training session has been shown to be sufficient for achieving competence in novice practitioners (emergency medicine physicians and senior medical students) for performing ultrasound-guided ilio-fascial blocks [19].

Even more complex surgical procedures have learning curves, which may not be exceedingly long. The learning curve for the Latarjet procedure, which is a complex procedure involving a bone transfer, has been estimated at about 22 cases [10]. Even the arthroscopic Latarjet, considered to be more technically difficult than the traditional open technique, has a learning curve between 20 and 40 cases [10,20]. The same is true of hip arthroscopy, generally considered to be one of the more technically difficult joints when it comes to arthroscopic surgery. A systematic review by Hoppe et al. found that a cut-off of just 30 cases demonstrated significant differences in patient outcomes [20].

Current procedures for the treatment of carpal tunnel syndrome

There exists a variety of techniques to perform carpal tunnel release, all with the common goal of releasing the transverse carpal ligament to decompress the median nerve within the carpal tunnel [1]. Traditional open CTR remains the commonly used surgical technique, involving a skin incision of typically 2-3 inches in length. The surgery is generally successful in appropriately selected patients, however, 10-20% of patients may have persistent symptoms, and many patients have delayed recovery due to the need to protect the wound during healing and complications such as pillar pain, which is the most common complication of traditional open CTR [21]. In addition, scar tenderness and/or sensitivity and a longer time away from work and other activities are concerns with open CTR [1,22].

To address some of these common concerns with traditional open CTR, mini-open and endoscopic CTR (ECTR) techniques have been developed in the hopes of minimizing soft tissue insult while achieving the same goals as traditional open CTR [23]. A meta-analysis of randomized controlled trials (RCTs) comparing mini-open with traditional open CTR demonstrated a faster return to activity and decreased rate of adverse events [24]. ECTR techniques typically have smaller incisions as compared to mini-open CTR (mOCTR), and some ECTR techniques place the incision at the level of the wrist instead of the palm. With regards to ECTR, the best available evidence has been mixed about the potential benefits of ECTR. A recent meta-analysis of RCTs has demonstrated that while there is a benefit in the early recovery period and fewer scar complications, this technique also carries a higher risk of nerve injury [21], likely due to the use of endoscopic instruments with a limited visualization of the surgical field.

Carpal tunnel release with ultrasound guidance

**Figure 2:**
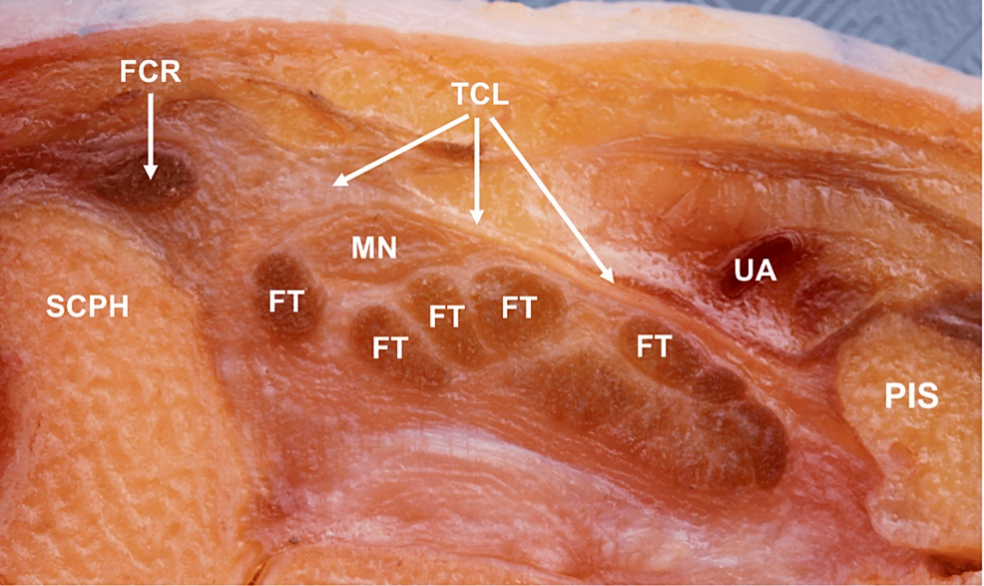
Anatomic cross-section in an unembalmed cadaveric specimen, demonstrating the proximal carpal tunnel region, defined by the scaphoid (SCPH) and pisiform (PIS) bones The transverse carpal ligament (TCL) spans between the scaphoid and pisiform, forming the roof of the proximal carpal tunnel. Within the tunnel are located the median nerve (MN) and nine flexor tendons (FT, not all tendons are labelled). During carpal tunnel release, the TCL is transected to create more space in the tunnel and reduce pressure on the median nerve. Also shown are the flexor carpi radialis tendon (FCR) and ulnar artery (UA), which lie outside of the carpal tunnel. Although the ulnar artery lies outside of the carpal tunnel, its close proximity to the TCL and the carpal tunnel renders it vulnerable to injury during carpal tunnel release procedures. Top = superficial, Left = radial Figure property of the authors

**Figure 3:**
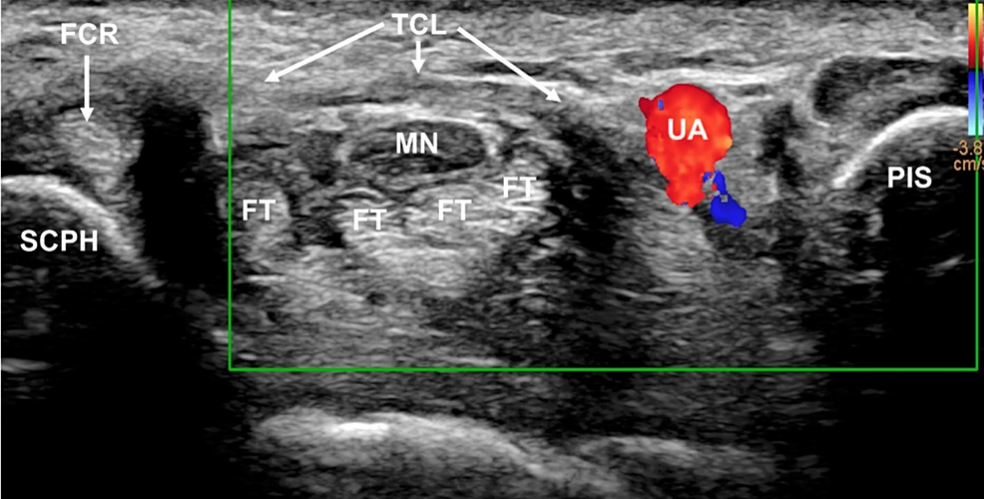
Correlative ultrasound image obtained with a 4-18MHz transducer in a live patient All anatomic structures seen in Figure 2 are visualized, demonstrating the wide field of view of ultrasound. This image was obtained with the Doppler ultrasound function being activated, which clearly identifies the pulsating ulnar artery. Label and orientation as the same as in Figure 2. Figure property of the authors

CTR-US represents an increasingly popular treatment for CTS [25], which may be able to provide the benefits of ECTR while avoiding the limitations of ECTR such as a reduced field of view [26]. Although CTR-US was first described as early as 1997 [27], recent advances in the quality, availability, and affordability of ultrasound technology have allowed this technique to become more commonly studied [6,25]. The primary advantage of CTR-US is the ability of the provider to identify all relevant anatomical landmarks despite being a minimally invasive technique [28]. During CTR-US, real-time ultrasound is used to identify important anatomic landmarks, including the thenar motor branch (also known as the recurrent motor) [29,30] and the superficial palmar arterial arch. A small entry point is then created in the distal forearm or wrist, and the transverse carpal ligament is transected under ultrasound visualization. The ligament may be probed to ensure complete release. The wound is then dressed, and sutures are typically not needed [22,25,31]. Figure 2 and Figure 3 show the surface and corresponding ultrasound images of the major anatomic landmarks for CTR-US, demonstrating the visibility that the US provides in this indication. An additional advantage of CTR-US is that it can be readily performed using only local anesthesia such as the wide-awake local anesthesia with no tourniquet (WALANT) technique. This promotes faster recovery, avoids the side effects of sedating medications, and allows the procedure to be performed in a procedure room setting, including the office [25].

Evidence to date has shown CTR-US to be a safe and effective treatment for CTS. An RCT comparing CTR-US to mini-open CTR found a significantly faster return to normal daily activities (~5 vs 25 days) and significantly faster improvements in Quick Disabilities of the Arm, Shoulder, and Hand (QuickDASH) scores [32,33]. A recent systematic review assessing the literature on CTR-US found that compared to open CTR, there is the potential for faster and easier post-operative recovery with this technique [6].

The learning curve of CTR-US

Ultrasound has demonstrated its accuracy in identifying and diagnosing CTS [33]. This comprehensive meta-analysis on the use of diagnostic US for CTS pooled the accuracy rates in 28 separate CTS diagnostic studies and showed sufficient diagnostic accuracy for CTS across this body of evidence. Not only has the US been investigated for its use in CTS diagnosis, but there is also a body of evidence specific to the learning curve of CTR-US from an interventional perspective.

As with any ultrasonographic technique, proficiency is highly dependent on the operator [34]. Thus, there have naturally been some questions about the learning curve associated with CTR-US. The interest in the learning curve associated with CTR-US is clear when examining the literature on the topic - full texts of 18 studies included in the most recent systematic review on CTR-US [6] could be accessed. Among these studies, 17 studies (94.4%) mentioned specifically which provider(s) had performed the procedures. The majority (15/17, 83.3%) specifically referenced the provider’s experience with the procedure and/or ultrasound. Despite this, most studies did not specifically quantify experience in terms of the number of procedures or ultrasounds performed, but rather in general terms (e.g. XX years of experience in interventional radiology). The providers included had a wide range of background training profiles, including radiology, physiatry, orthopedic surgery, and hand surgery [6].

Dekimpe et al. analyzed the learning curve in both a senior and junior radiologist in a cadaveric model. The senior radiologist had five years of experience in musculoskeletal interventional radiology while the junior radiologist had two years of internship in diagnostic radiology with no interventional experience. They demonstrated that the senior and junior radiologists required only three and four attempts, respectively, before demonstrating high proficiency and accuracy at the task [35]. Another cadaveric study by Mittal et al. demonstrated that two fellowship-trained physiatrists who were experienced with ultrasound but not CTR-US specifically rated the difficulty of the procedure as a 2.6 on a 5-point scale [36]. Furthermore, Hebbard et al. analyzed CTR-US in 10 cadaveric specimens; while quantifying the learning curve was not an objective of the study, they did observe a learning effect over the course of the study with later procedures being performed more quickly (about 15 minutes to complete) [37]. This body of literature helps elucidate the learning curve CTR-US, as ample evidence exists on the specific anatomic landmarking required, as well as the anticipated learning curve observed in both junior and senior clinicians.

## Conclusions

Overall, CTR-US holds promise as a technique for treating the most common compressive neuropathy. It allows the provider to visualize all critical structures of the wrist while minimizing incision size and allowing for the procedure to be completed using a minimal set of low-profile instruments, which can potentially help minimize the risk of neurovascular injury. Given the fact that CTR-US can be performed in a variety of settings, including the office, and is the minimal equipment needed to perform each case, CTR-US also carries the potential for considerable economic savings. The learning curve for other ultrasonographic procedures in musculoskeletal medicine has been demonstrated to be relatively short. Specifically, the skills required for CTR-US are attainable by practitioners and trainees at various levels of experience, both in general and with US specifically. Thus, concerns with the learning curve associated with CTR-US should not be prohibitive to further development and evaluation of this technique. Further evaluation of CTR-US is needed in high-quality clinical studies to compare its efficacy, safety, and cost with traditional open and endoscopic CTR.
